# Wearable Monitoring Devices for Biomechanical Risk Assessment at Work: Current Status and Future Challenges—A Systematic Review

**DOI:** 10.3390/ijerph15092001

**Published:** 2018-09-13

**Authors:** Ranavolo Alberto, Francesco Draicchio, Tiwana Varrecchia, Alessio Silvetti, Sergio Iavicoli

**Affiliations:** 1 Department of Occupational and Environmental Medicine, Epidemiology and Hygiene, INAIL, Via Fontana Candida 1, Monte Porzio Catone, 00078 Rome, Italy; f.draicchio@inail.it (F.D.); al.silvetti@inail.it (A.S.); s.iavicoli@inail.it (S.I.); 2 Department of Engineering, Roma TRE University, Via Vito Volterra 62, 00146 Rome, Italy; tiwana.varrecchia@uniroma3.it

**Keywords:** standardized biomechanical risk assessment methods, instrumental-based biomechanical risk assessment, wearable sensors, sEMG, IMUs, hand-held dynamometers, grip force sensors

## Abstract

*Background*: In order to reduce the risk of work-related musculoskeletal disorders (WMSDs) several methods have been developed, accepted by the international literature and used in the workplace. The purpose of this systematic review was to describe recent implementations of wearable sensors for quantitative instrumental-based biomechanical risk assessments in prevention of WMSDs. *Methods*: Articles written until 7 May 2018 were selected from PubMed, Scopus, Google Scholar and Web of Science using specific keywords. *Results*: Instrumental approaches based on inertial measurement units and sEMG sensors have been used for direct evaluations to classify lifting tasks into low and high risk categories. Wearable sensors have also been used for direct instrumental evaluations in handling of low loads at high frequency activities by using the local myoelectric manifestation of muscle fatigue estimation. In the field of the rating of standard methods, on-body wireless sensors network-based approaches for real-time ergonomic assessment in industrial manufacturing have been proposed. *Conclusions*: Few studies foresee the use of wearable technologies for biomechanical risk assessment although the requirement to obtain increasingly quantitative evaluations, the recent miniaturization process and the need to follow a constantly evolving manual handling scenario is prompting their use.

## 1. Background

In recent years, wearable sensors have been used for quantitative instrumental-based biomechanical risk assessments in the prevention of work-related musculoskeletal disorders (WMSDs). Previously, in the attempt to reduce the risk of WMSDs while handling materials, handling people in the healthcare sector or while maintaining fixed postures, several methods have been developed, accepted by the international literature and used in the workplace. These approaches have without doubt facilitated prevention activities during the last decades by improving occupational health and safety of people at work but, on the other hand, need a significant update based on two main aspects. First, the standardized methods commonly used for biomechanical risk assessment are still mainly based on observational and subjective approaches [1,2,3,4] and don’t include instrumentation-based tools. Second, the recent widespread use of robots, automation and mechanization in industry for the reduction of the physical effort has modified manual handling work activities. One of the key technologies driving this epochal change, the human-robot collaboration (HRC) technology [5,6], is invading several areas of the industry and small-medium enterprises. The nascent nature of HRC in the workplace conceives the safe coexistence and interaction of workers and robots within the same environment allowing a significant transformation of the current static automation paradigms into adaptive, flexible and reconfigurable ones. In particular, the presence of the most advanced remotely controlled robot, occupational collaborative robots [7] and wearable trunk and upper-limb exoskeletons [8,9,10,11,12] will assist more and more workers in performing their tasks reducing their exposure to the associated physical demands.

In view of this new workplace setup there are some questions to ask: are the standardized biomechanical risk assessment methods able to take into account all these new factors? Are the most recent electronic wearable technologies used for biomechanical risk assessment? And again, can they be considered the answer to the aforementioned advanced “Industry 4.0” manufacturing solutions? The authors of this review propose that while advances in wearable wireless sensor networking and ubiquitous computing have paved the way for new possibilities in sport performance measures [13,14,15,16] and clinical applications [17,18,19,20], today their potential for biomechanical risk assessment is still largely underexploited and the state of the art lags dramatically behind the expectations. The hypothesis underlying this review is that the most innovative wearable technologies and electronic smart devices such as smartphones and tablets may improve the biomechanical risk assessment by adapting it to all the work conditions and overcoming the limits of the current standardized methods. For instance, intelligent work environments [21,22] may represent the new scenario in which smart wearable sensors with computational capabilities and network connection are sensitive, responsive, adaptive and transparent [23] to workers’ movements allowing online, real-time monitoring of work activities.

Thus, these devices, without interfering with the typical movements performed by workers at the workplace thanks to the miniaturization process and wireless protocols, would allow the estimation of biomechanical risk in real-time providing a direct feedback to the end-user who would be constantly monitored directly at work. In this way, the workers could modify their movements during the execution of work tasks thereby reducing and preventing their exposure to the risk of WMSDs.

To shed light on this issue, the aim of this review was, through a literature research (Section 2), to describe recent implementations of wearable sensors for quantitative instrumental-based biomechanical risk assessments in the prevention of WMSDs. To do this, we have provided:

- A brief description of the WMSD problem and of some standardized methods used for biomechanical risk classification, with their respective strengths and weakness (Section 3.1).
- An explanation of how wearable sensors work and measurements are performed, with particular attention to inertial measurement units (IMUs), hand-held dynamometers and grip force devices, and surface electromyography (sEMG) sensors (Section 3.2).
- A description of quantitative instrumental-based biomechanical risk assessment methods, which have proved themselves significant for physicians, ergonomists and researchers. These proposed tools have been analyzed for: (i) direct instrumental evaluations [24,25,26] providing real-time measures of risk of exposure, requiring simple hardware setup and allowing easy analysis and interpretation of data by workers (Section 3.3); (ii) rating standard methods for biomechanical risk assessment (Section 3.4).
- Finally, a discussion covering current issues, future challenges and limitations is reported in Section 4.

## 2. Materials and Methods

### 2.1. Variable of Interest

The variable of interest in this systematic review [27] was “wearable technologies for quantitative biomechanical risk assessment in work activities (i.e., lifting tasks, pushing and pulling, repetitive work handling people manual tasks in an industrial environment) able to minimize the disturbance caused by instrumentation to the user”.

### 2.2. Literature Search Strategy

The literature search was performed from date of inception until 1976 on the following selected databases: Scopus, Web of Science, PubMed and Google Scholar. Weekly updates were performed until 7 May 2018. The following keywords concerning wearable devices, biomechanical risk assessment and work activities, were identified and combined: instrumental-based biomechanical assessment methods, manual lifting, handling people, pushing and pulling, repetitive moments, wearable sensors/devices/technologies, movement analysis, kinematics, kinetics, sEMG, IMUs, hand-held dynamometers and grip force sensors.

Electronic searches were performed by one author (R.A.), who screened all potential titles, abstracts, and if needed, full-texts for eligibility. The reference lists of all the selected articles were also scanned to identify other eligible articles.

### 2.3. Review Process

**Figure 1:**
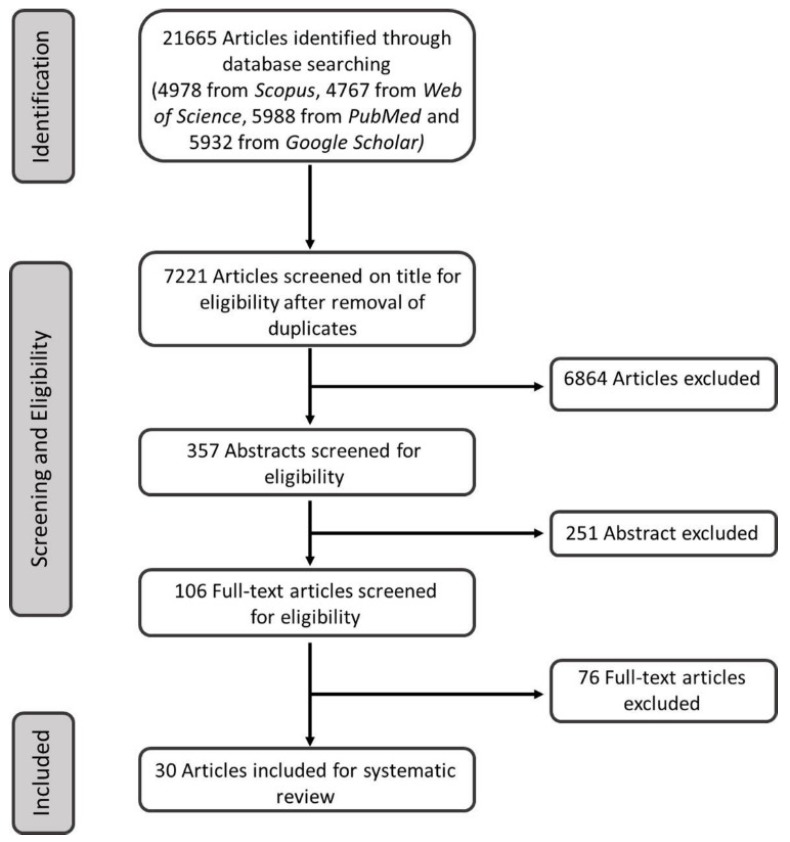
Flow-chart according to the different phases of the systematic review as proposed by PRISMA.

The study was conducted using the systematic review method proposed by the Preferred Reporting Items for Systematic Reviews and Meta-Analysis (PRISMA), as shown in Figure 1. The search was limited to papers in journals, chapters of books and periodicals, conference proceedings and Ph.D. dissertations. For all the duration of the screening selections were based on the significance of the recognized articles regarding the matter of the review. Three independent reviewers (R.A., T.V. and A.S.) assessed titles and abstracts of the articles. The full text reading was done when titles and abstracts offered satisfactory information. Disagreements among reviewers were resolved by scheduling dedicated consensus meetings of all authors.

## 3. Results

From the database search, after removing duplicates, 7221 references were retrieved and screened for eligibility based on their titles (Figure 1). Following that, 357 abstracts and 106 full text articles were assessed for eligibility. Articles were excluded due to the facts that wearable devices were not used in biomechanical risk assessment or they were not used in work activities, biomechanical risk assessment methods were not instrumental, there was no instrumental assessment of work activities, movement analysis (kinematics, kinetics, sEMG, IMUs, hand-held dynamometers and grip force sensors) was not in the work activities or instrumental approaches were not used. A total of 30 articles were finally included in this review.

### 3.1. Work-Related Musculoskeletal Disorders (WMSDs)

WMSDs are widespread in many jobs and a constantly growing concern for health and safety workers and for manufacturing system productivity [2,3]. The annual incidence of WMSDs in the industrialized world accounts from a third to a quarter of all occupational diseases, making these disorders the most expensive form of workplace disability [28,29,30,31,32,33,34,35,36,37,38,39,40,41,42].

Among WMSDs, work-related low-back disorders (WLBDs) and upper limb work-related musculoskeletal disorders (UL-WMSDs) are the most common, with a 12-month worldwide prevalence of almost 18% [43,44,45,46,47,48,49,50,51] and ranging from 12% to 41%, respectively [52]. In particular, the proportion of the population exposed to ergonomic risk factors for WLBDs is 87% according to the Global Burden of Disease 2016 [53]. Furthermore, WMSDs account for 64% of the total number of reported occupational disorder cases [54].

WMSDs occur when the spinal load exceeds tissue tolerance [55,56], when the central nervous system co-activates antagonistic trunk muscles in the attempt to stabilize the trunk in presence of excessive external loads [57] or when the working tasks lead to local muscle fatigue [58,59,60,61]. For this reason, the relationship between manual handling tasks and musculoskeletal health is considered multifactorial [62] although task features, individual biomechanical and physiological factors accompanied by altered motor control mechanisms represent the main determinants [63]. Critical working tasks are predominantly manual lifting and patient handling, pushing and pulling and repetitive activities, awkward and/or sustained postures and prolonged sitting [50,64,65,66,67,68,69,70,71,72,73,74,75,76].

The international standards 11228-1, 2 and 3, 11226, TR 12295 and TR 12296 [77,78,79,80,81,82] accept and list the methods able to detect occupational physical risk factors and to evaluate the usefulness of ergonomic interventions. These methods consider several indices to be measured, from motion amplitude and frequency to exerted force [2].

The strength of these traditional approaches for biomechanical risk classification which can be used in a wide range of professional conditions, is based on their inexpensiveness and non- invasiveness. On the other hand, these methods have some weaknesses, mainly due by their observational nature and subjectivity related to the practitioner’s expertise [1,2,3,4]. In most cases the worker’s behavior is evaluated on pro-forma sheets either while observing in the field or replaying videos, an approach considered inaccurate, imprecise and time consuming [2,83,84]. Furthermore, the scientific literature highlights equations and parameter restrictions, with insufficient accuracy, unrepeatability and unreliability [4,85,86]. These concerns are mainly due to the necessity of assignment of scores to each risk determinant, such as upper and lower limb joint angles and range of motions, loads displacement, forces, work cycles, frequency of actions, forces and recovery times [85,87,88,89,90,91,92,93,94].

Accurate and precise results could be better achieved by means of modern measuring devices, facilitating experts’ diagnostics [2]. In recent years, instrument-based techniques designed based on current technological advances and performing direct measurements by using sensors attached to workers’ bodies have been developed (see Table 1) and are now able to capture some or all of the parameters needed in the computation of the risk level. These instrumental and quantitative sensor-based tools might greatly increase the accuracy of these methods in ways that were not previously available [25,69,70,95] and widely reduce the time that an expert needs to carry out the same assessment manually. Finally, the use of automatic online tools would give a meaningful evaluation by gathering posture, kinematic, kinetic and muscular activity data in assessing WMSDs risk.

### 3.2. IMUs, Hand-Held Dynamometers and Grip Force Devices, sEMG Sensors: How They Are Made and Measure

Movement analysis systems allow, with a high accuracy and acquisition frequency, the quantification of motor functions, motor abilities, pathological conditions, compensatory motor strategies and improvements due to rehabilitation treatments and ergonomic interventions. However, these systems can be easily used only within the laboratory and more difficult in the field. This difficulty has led to the development, in the last decade, of accurate and reliable wearable human body sensor-based tools for easy human motion analysis directly in the workplace. The main factor allowing the abovementioned use has been, without doubt, the miniaturization of devices which has allowed huge benefits over traditional approaches. Other factors are wireless connectivity, light weight, small-size, low power consumption, portability, low-cost, comfort, and the possibility to monitor subjects remotely and to provide feedback to the end-user [19,96,97,98,99,100,101,102].

**Figure 2:**
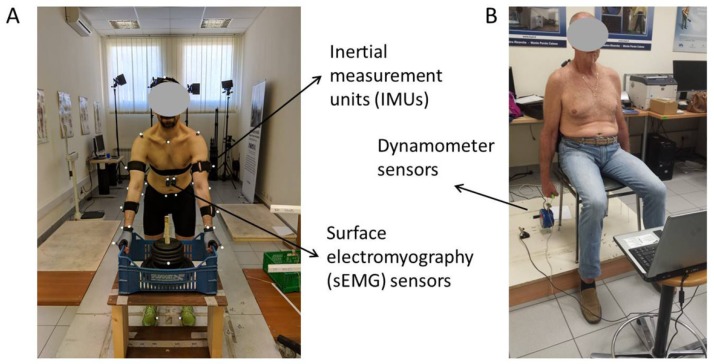
Sensors in ergonomics application: (**A**) Inertial measurement units (IMUs) and electromyography (sEMG) sensors used in lifting acitivities; (**B**) Dynamometer used before and after repetitive work.

Among wearable human body sensors, inertial measurement units (IMUs), dynamometers and surface electromyography (sEMG) sensors (see Figure 2) allow a detailed estimation (compared to traditional observational methods) of kinematics, kinetics and muscle behaviors without interfering with the typical movements performed by workers in the workplace [103,104].

In experimental settings, IMUs, dynamometers and sEMG sensors are placed and fixed on the appropriate body segments to measure joint angles, forces and muscle behaviors, respectively. All the sensors are commonly synchronized for data alignment in time [105]. Connection is always performed by implementing one of two wireless protocols: Wi-Fi or Bluetooth. The former has an increased power consumption, but a greater transmission speed and distance with respect to the latter. The sample frequency of these sensors varies between 50 and 1000 Hz while the minimum number of bit is 12 [106].

#### 3.2.1. IMUs

IMUs allow the measure of orientation, position, velocity and accelerations of each investigated segment and whole body posture. The term “inertial” comes from the fact that these sensors use the inertia principle: the acceleration can be related to the resistance to move (inertia) of a free mass accelerated by an external force or torque. In the literature, the type of sensors used ranges from uniaxial to triaxial accelerometers, gyroscopes and magnetic sensors [99]. Usually three orthogonal accelerometers and three orthogonal gyroscopes are embedded within the probe to measure linear acceleration and angular velocity, respectively, along three orthogonal axes. Angular displacements are obtained from numerical integration of the angular velocity while linear velocity and displacement are estimated from first and second numerical integration of linear accelerations. IMUs can also embed tri-axial magnetic sensors although their use turns out to be more critical in the workplace in presence of electromagnetic fields.

#### 3.2.2. Hand-Held Dynamometers and Grip Force Sensors

Hand-held dynamometers, already described at the beginning of the previous century [107], are simple devices placed between a fixed place and the subject’s body part to assess the isometric muscle (or muscle group) strength relevant as outcome measurements in studies evaluating changes in the functional status of joints, lower and upper extremities and trunk [108,109,110,111,112,113,114]. These devices are considered highly reliable [115], easy to use, portable, inexpensive and compacts if compared with isokinetic systems [112]. Besides hand-held dynamometers, forces are also recorded by using superior grip dynamometers although they can only be used for given hand sizes. Other critical issues related to grip dynamometers are the inability to measure forces from multiple fingers simultaneously, difficulty of use, low accuracy of grip force measurements and the difficulty to design appropriate handle shapes [116,117,118,119,120]. The measure of the grip force is also provided by instrumented gloves (i.e., equipped by force sensitive resistors) or by force sensor mats applied to handles [73,121,122,123,124,125,126]. Instrumented gloves remove the need for the handle to be instrumented but disturb grasp interaction [127]. Furthermore, force sensor mats embedded within gloves acquire only normal forces, require calibration and may shift during measurements. In order to overcome these limitations multi-dimensional grip dynamometers have been developed to adapt to a wide variety of handle sizes and geometries allowing a continuous measure of fatigue and forces [119,127,128]. Recently dynamometers able to measure both angle and force with high levels of sensitivity and inter-examiner reliability have been developed [129,130,131,132]. Finally, it is interesting to also report haptic tools which consist of physical bendable strips allowing users to manipulate and apply deformations to digital surfaces and to move and rotate virtual objects. Such device allows a continuous, free hand contact on a developable strip bent allowing to industrial designers and stylists to perform an effective assessment of the aesthetic quality of the shape of new products and also its modification, directly on the digital prototype without the need to construct a physical prototype [133,134,135].

#### 3.2.3. sEMG Sensors

sEMG provide the measure of electrical activity (on the skin) of the muscles involved in the movement. Single- or double differential bipolar sEMG performed by using wet electrodes is widely and easily used in ergonomics for research activities and directly at the workplace [136,137,138,139,140,141]. sEMG allows the calculation of a lot of parameters regarding muscle behavior such as, among other features, the “activation timing” [142,143,144,145,146,147,148,149,150], the amplitude (maximum values, average rectified values or ARVs, root mean square or RMS) [137,146,151,152,153,154,155] and co-activations [57,66,156]. Multi-channel sEMG performed by means of linear and two-dimensional electrodes arrays (high-density sEMG) allows instead the estimation of the motor unit action potential analysis [157,158,159,160,161], the estimation of the local muscle fatigue (the myoelectric manifestation of muscle fatigue) [60,138,162,163,164,165,166,167] and the analysis of the instantaneous potential maps [160]. In particular the local muscle fatigue is estimated by measuring the decrease in the fiber conduction velocity [168,169] which imply a scaling of the sEMG signal power density towards the lower frequencies and an increase its amplitude [170,171,172].

### 3.3. Direct Instrumental Evaluations

In this section of the results we report papers whose aims were to propose instrumental-based tools for biomechanical risk classification without using measured/calculated indices as input to standardized methods. Instrumental approaches based on wearable sensors have been used to classify lifting tasks into low and high risk categories. In a very recent study [173], IMUs and sEMG sensors have been used to monitor trunk inclination and trapezius and erector spinae muscle activity, respectively, during the execution of several types of lifting tasks with different weights, horizontal distance and technique executed by male office workers. The method proposed in this study allows, with an acceptable accuracy, the automatic identification of the risk levels associated with the lifting activities. Indeed, the lifting tasks were characterised by a feature vector composed of either the 90th, 95th or 99th percentile of sEMG activity level and trunk inclinations during the task. Linear Discriminant Analysis and a subject-specific threshold scheme were applied and lifting tasks were classified. The authors of this study highlighted how the strength of this study lies on its objective instrumental approach based on subject-specific thresholds and on the possibility to complement the current standardized approaches usually used to detect biomechanical hazardous.

Another recent kinematic-based lifting tool has been designed by monitoring several lifting tasks with growing lifting index (LI) computed by the revised NIOSH lifting equation [174]. Kinematic data allowed the calculation of a mechanical lifting energy consumption (LEC) index which proved to be significantly growing with the LI, discriminating all the risk condition pairs and well correlating with compression and shear forces that determine injuries at the L5-S1 joint. The findings of this study suggest a potential use of IMUs-based lifting tools in indoor and outdoor work environments for risk estimation.

Furthermore, muscle coactivation has deeply been investigated [66] because it, being a neuromuscular pattern needed to stabilize the trunk [57], represents one of the causal pathways for WLBDs. The behavior of the cervical and lumbar spine has also been investigated in complex multiplanar dynamic motions including lifting and pushing [175,176]. sEMG has been used to develop a sEMG-based multi-muscle coactivation index that resulted usable to continuously assess the neuromuscular effort and significantly sensitive to several factors. In particular the higher the speed, complexity of the motion and head control are, the higher the coactivation index value is. Also, in this case this simple approach has been proposed to be used for ergonomic assessments.

Another tool developed to calculate the simultaneous activation of trunk muscles is the time-varying multi-muscle co-activation index (TMCi) which includes a sigmoid-weighting factor dependent on relative differences between muscles that do not rely on a priori definitions of agonist or antagonist behavior [156]. This index was evaluated during the execution of lifting task in controlled conditions considering trunk muscles [156]. It has been shown that heavier lifting conditions resulted in higher TMCi values and that significant correlations exist between the TMCi and other agonist–antagonist methods. The same experimental setup used for LEC calculation [174] allowed to understand that also simple sEMG parameter values (i.e., ARV and max), besides TMCi, increased under heavier lifting conditions [177].

Moreover, sEMG data were also used in a study to implement tools based on an artificial neural network [178]. In this study sEMG features (i.e., max, ARV, mean and median frequencies) were evaluated during the execution of lifting tasks starting from the trunk muscles. Then, these features were used as input variables of artificial neural network for the prediction of WLBDs. The results show that sEMG time and frequency features are significantly related to lifting index for specific trunk muscles. Furthermore, the findings show that a tool based on these machine-learning techniques and sEMG feature, choosing a proper combinations of input features and a right network architecture, can lead to an improved biomechanical risk classification. Moreover, the authors concluded that the possibility to implement the integrated approach on electronic smart devices (smartphones, phablets, tablets and smartwatches) would allow a simplified analysis of biomechanical risk at workplace.

Manual lifting has also been assessed by using muscle fatigue estimation. In a recent review regarding this issue a list of methods was given, though the authors concluded that there are still many gaps to be filled and further studies are needed to find better fatigue indices and improved techniques [179].

Besides lifting activities, wearable sensors have been used for direct instrumental evaluations in handling of low loads during high frequency activities. For instance, local myoelectric manifestation of muscle fatigue estimation [160,161,180] has been estimated in several conditions to investigate several groups of workers. In particular it was used to investigate: (i) the biomechanical exposure of younger and older groups [181,182]; (ii) changes in fatigability in jobs with and without pause [166]; (iii) upper limb and trunk muscles in simulations of assembly tasks and in different light levels of repetitive work [162,163,164,165,167,182,183,184,185]. Further experiments have been designed and performed in a recent study of Ranavolo and colleagues [58] in which the association between the local elbow flexor muscles fatigue and physical demand has been investigated. The findings of this study showed that local muscle fatigue estimated by using miniaturized sEMG sensors placed on the brachioradialis muscle is a promising index because of its sensitivity to the risk classes. Kinematic and sEMG assessments have also been performed in biomechanical evaluation of supermarket cashiers before and after a redesign of the checkout counter, in analysis of post office employees’ workstations and in manual handling on a supermarket greengrocery shelf [186,187,188].

Finally, the usefulness of wearable sensors has also been investigated in many work tasks which require intensive and repetitive production of forces on the upper extremities in manipulating external loads, wrists, palms, fingers and tendons [189,190,191]. In these cases, the role of wearable sensors, in most cases hand-held dynamometers devices, is to measure the normal and shear forces created between fingers and handles to assess muscle integrity and to determine the level of any strength deficits [192,193] associated to clinical physical examination tests (i.e., the diagnosis of shoulder pain [194]). A wearable, unobtrusive, wireless and accurate system (Activity Tracking with Body Area Network) has been designed to operate autonomously to quantitatively measure the postures and body motions of workers [2]. This system is meant to be used by workers to autonomously monitor themselves on actual job sites over long periods of time. Different working processes in a wood workshop have been evaluated by using three accelerometers and two microphones and by correlating the worker’s motion and frequency and intensity of sounds [195]. IMUs were also used in several work activities such as car assembly, hammering, screwing and drilling [196,197]. In construction activities IMUs [198,199,200] and sEMG has been used to monitor lifting and holding loads activities to detect potential sources of WMSDs at neck [201] lower back levels [202].

### 3.4. Risk Assessment in the Context of Rating of Standard Methods

As done for direct instrumental evaluations, in this section tentative ratings of standard methods using wearable technologies are analyzed. An innovative “on-body wireless sensors network”-based approach for real-time ergonomic assessment in industrial manufacturing has been proposed by Vignais and colleagues [84]. The sensor network was composed by IMUs and goniometers and the body posture (joint angles) was assessed by using a ten rigid segment, twenty degrees of freedom biomechanical model. IMUs were placed bilaterally on the upper arm and forearms, on the head, trunk (on the chest) and pelvis (on the sacrum). Goniometers were placed on the hands and forearms to measure wrist motions. Angle values were used as input within the Rapid Upper Limb Assessment (RULA) method, whose global and local scores were continuously computed by a mobile processing unit (a standard laptop) and fed back to the user via a see-through head-mounted display.

Moreover, a real-time body sensors network composed by IMUs and sEMG sensors has also been used in real-time to monitor workers by measuring muscular efforts and postures (upper limbs have been modeled as a 7-DoF kinematic chain) for WMSD prevention according to the RULA index and the Strain Index (SI) [3]. An interesting index considered for this tool is the percentage of time spent in every RULA score range by every worker, considering the whole experiment duration. The accuracy, expressed as the number of correct assessments (with respect to those performed by two human evaluators) of the system and the number of cycles, was 95% for RULA and 45% for SI, indicating that the body sensor network is able to give a RULA score estimation congruent to the one given by the human evaluators. As far as the SI score is concerned, the system gives a score congruent to the evaluators’ evaluation in almost the 50% of the cases.

sEMG has also been used for complementing the RULA scoring system [203] and as an alternative to the visual inspection according to the BORG scale. It is in fact demonstrated that the two assessments are strongly correlated [87]. An example of the latter application has been studied by Cabeças [204] where sEMG was used as an alternative to observational methods in computing the SI score. The authors concluded that, once appropriate trigger levels for the muscular activation are defined, sEMG is a valid alternative to visual inspection in SI computation. This is true in particular when efforts are not clearly associated to hand/wrist movements and when non-cyclical high-frequency activities are assessed.

### 3.5. Main Findings

Among the 30 studies included in the review (Table 1), IMUs were used only in five [3,58,84,174,175], and dynamometers were used only in four [58,182,194,205], while sEMG sensors are used in 27 studies [3,57,58,65,66,87,156,162,164,165,166,167,173,175,176,177,178,179,182,183,186,188,201,202,203,204,205].

The results of these studies are mainly based on kinematic [3,84,173,174,183,186,188], kinetic [58,177,182,194,205] and sEMG [3,57,58,65,66,87,156,162,164,165,166,167,173,175,176,177,178,179,182,183,186,188,201,202,203,204,205] data. Among the most investigated indices there are those sEMG-based. In particular, the most investigated indices are “muscle fatigue” (in nine studies [58,162,164,165,166,167,182,183,205]) and “muscle coactivation” (in seven studies [57,65,66,156,175,176,177]).

Instrumental evaluations were performed in 13 studies [3,58,84,87,156,173,174,177,178,186,188,203,204], muscle fatigue [58] by using kinematic data (i.e., joint angles [84] and range of motions [186,188], posture and more complex kinematic indices [3,175], trunk inclination [173]), kinetic data (i.e., compression and shear forces at the L5-S1 joint [174,177]) and sEMG data (i.e., time, amplitude and frequency parameters [3,87,173,177,178,186,188,203,204], muscle fatigue [58] and coactivation [156,177]).

In these 13 studies, the most investigated working tasks are lifting tasks [156,173,174,177,178] and manual handlings of low loads at high frequency [58,186,188]. As regards lifting tasks, all the results of these studies show that kinematic [173,174] and sEMG-based [156,173,177,178] indices significantly grow with the LI discriminating all the risk condition pairs. Moreover, when a correlation analysis has been performed, results show a good correlation between kinematic and sEMG-based indices and compression and shear forces [174,177]. Moreover, only one study [178] used machine-learning techniques and sEMG features. Results of these studies led to an improved biomechanical risk classification. As regard the studies regarding the manual handlings at low loads at high frequency, the results show that sEMG indices were sensitive to the risk classes [58,186].

Many of these studies investigated specific work activities [3,84,87,186,188,204]: handling of low loads at high frequency on a redesigned checkout counter [189], laparoendoscopic single-site surgery [203], real-life operations of super-market cashiers [3], manual tasks in an industrial environment [84], sawmill work [87], manual handling on a supermarket greengrocery shelf [186], cleaning activities [204]. The results of these show that kinematic [3,84,87,186,188] and sEMG [3,87,186,188,204] data could be used in the risk assessment in work activities.

## 4. Discussion

In the attempt to reduce the risk of work-related musculoskeletal disorders several methods have been developed, accepted by the international literature and used in the workplace. In the last years, the most innovative wearable technologies and electronic smart devices, without interfering with the work activities performed by workers, have been introduced to improve the biomechanical risk assessment adapting it to all the work conditions and overcoming the limits of the current standardized methods. Indeed, these devices allow the estimation of biomechanical risk in real-time providing a direct feedback to the end-user who would be constantly monitored directly while at work.

In this review, we report on recent implementations of wearable sensors for quantitative instrumental-based biomechanical risk assessments in the prevention of WMSDs. Their use is desirable also in view of the concerns expressed within a recent article [206] regarding technical ISO standards on ergonomics and physical workloads.

In this “discussion paper” the authors underlined, among the others, how: (i) the production of these standards differed substantially from evidence-based practical guidelines; (ii) it is not clear why the ISO subcommittee preferred one method of risk assessment over others; (iii) some statements in ISO 11228 series appear to be based on personal opinions and in contrast with scientific evidence from the literature; (iv) ISO standards are an effort by a self-identified committee of interested people to agree on “how something should be made” in order to facilitate exchange of goods, services, or other similar endeavors; (v) ISO standards should be used with caution. For these reasons instrumental-based tool will play an increasingly important role in both direct evaluations and in the rating of standard methods, also in consideration that several factors implying work-related musculoskeletal disorders interact at the same time. Therefore, it will be crucial to monitor all of them by using more than one method at the same time ensuring a more thorough evaluation of risk factors. On the other hand, a lot of attention must be paid because the use of more than one method can rapidly lead to unacceptably high costs for the practitioner, both from a time and money viewpoint [207,208].

In this context, the technologies accredited to be used are without doubt inertial measurement units (IMUs), instrumented gloves and surface electromyography (sEMG) sensors, although other new tools are appearing in research laboratories and the workplace. Among these, smart footwear-based wearable systems [209] will surely be useful because they will permit, by recording ground reaction forces through integrated tri-axial force sensors, an inverse dynamics analysis [210,211,212]. For their simplicity, vision-based tracking systems are also potentially useful for the rating of standardized methods as proposed for assessing the movements of workers within quick exposure check tools [83]. Wearable miniaturized sensors can monitor workers’ motor behavior if individually placed on the body segments or embedded in elastic suits. The latter use is also the most probable because the research activity is working fast on the development of artificial muscles, materials able to reversibly contract, expand, and rotate due to an external stimulus [198,213,214]. These devices, that can be enriched by several material characteristics, textile layers, elastic components, diagonal and lateral seams and pneumatic mechanisms [215] are envisioned as actuators for silent, soft and compliant assistive devices [216] acting as force multiplier systems by helping workers to reduce their effort. These suits/devices can also embed miniaturized sensors which will also serve for their control through, for instance, effective feedforward anticipation mechanisms. Furthermore, numerous devices have been developed to support the trunk during dynamic lifting tasks. sEMG will allow the detection of the early preparatory muscle activities to classify muscle loading and to initiate appropriate device activation. It has been shown that preparatory muscle activity can be leveraged to identify the intent to lift a weight up to 100 ms prior to load-onset [217]. The reduction of the effort will also be guaranteed by highly adaptive production processes.

Although the use of new innovative technologies for biomechanical risk assessment is only at the beginning (see Table 1), the literature shows that these instrumental approaches could be used to classify lifting tasks into low and high risk categories. The reported studies used wearable sensors, such as inertial measurement units, dynamometers and surface electromyography sensors, for biomechanical risk assessment in different work activities: lifting tasks [58,65,66,173,176,177,201] manual tasks in an industrial environment [84,164,183], manual handling [174,186], work in supermarket [3,188], repetitive work [165,166], cleaning activities [204], surgery work [203].

The results of these studies show that the indices used for the instrumented-based approaches are proved to correlate with the variables that determine the injuries. Particularly, two of the most promising indices/approaches proposed in literature for a work activity such as manual lifting, are the multi-muscle coactivation index [65,66,156,177] and machine-learning techniques based on sEMG features [178] while for this work activity, the literature shows that the fatigue indices need further elaboration [179]. Probably the most critical factor in lifting activities is the frequency of the lifting action which at the current status cannot be determined by instrumental methods [177]. It is necessary to be able to parameterize the risk levels associated to it. On the other hand, frequency of the actions is taken into consideration in work activities concerning the repetitive movement when the assessment is performed by using fatigue estimation before and after the work activity [58]. In some studies, wearable technologies are used for the rating of the standard methods (see Section 3.4). In these studies, the observed discrepancies between the evaluators can be due to several factors related to both the human and the procedural sides [203]: artifacts in the sEMG signals, difficulties in performing his real maximal voluntary contraction for the muscles considered, and underestimation by the human investigators of the actual efforts exerted by the workers.

From a technological point of view IMUs do not suffer from such limitations but if a high number of units is required for whole-body bio-mechanical studies in ergonomics, a high data transfer time could be required with both the Wi-Fi and Bluetooth protocols. Furthermore, IMUs fail to precisely measure translational motion and suffer from drift. Finally, IMUs can fail in the presence of magnetic fields in the workplace if they have embedded magnetic sensors. As regards limitations associated to sEMG, crosstalk muscle signals, electrode–skin impedance, noise and problems related to the electrode location, size, configuration and distance are the main critical factors [218]. To optimize the sEMG measures it is essential to use reference books such as the “*Atlas of Muscle Innervation Zones*” [219]. For both IMUs and sEMG sensors the energy consumption and the consequent battery discharge do not seem to be problems anymore, thanks to the long life of the most recent batteries. Two main limitations are ascribable to dynamometers: forces are commonly measured in only one direction and the form factor of the handles is not characteristic of most handles encountered during everyday work activities [220,221,222].

Finally, the heterogeneity in experimental procedures of the articles included in this review such as in working tasks, body segments, muscles and instrumental-based indices, has not allowed a valid complex statistical analysis combining the results as in other systematic reviews. For this reason, we have avoided a statistical pooling and summarized the data narratively [222,223,224,225]. The lack of a meta-analysis can be considered a limitation of the study.

## 5. Conclusions

The analysis of the papers reported in this review sheds light on the fact that still too few researchers foresee the use of wearable technologies for biomechanical risk assessment although the requirement to obtain increasingly quantitative evaluations, the recent miniaturization process and the need to follow a constantly evolving manual handling scenario is prompting their use. Therefore, the use of new innovative technologies for biomechanical risk assessment is only at its initial stage, but the authors of this review believe that this process is unstoppable, as it is happening in all the other areas of medicine and beyond. Obviously, it will be necessary for any validation to follow evidence-based medicine/policy/legislation multistep scientific approaches by designing rigorous laboratory and epidemiologic studies, by replicating them by independent research groups and by systematically evaluating them through transparent review processes. We are however convinced that, even if such use should fail in ergonomic practice, the huge knowledge that will derive from its experimentation will allow the optimization of the current standardized methods or the developments of the new ones.

## Figures and Tables

**Table 1:** Instrument-based techniques designed on current technological advances and performing direct measurements by using sensors attached to the workers body. M and F indicate male and female respectively. IMUs: inertial measurement units; sEMG: surface electromyography.

Wearable Sensors	Author (Year)	Sample	Work Activity	Body Part Assessment	Aim	Findings	Quantitative Data
IMUs	Vignais et al. (2013) [84]	12 M	Manual tasks in an industrial environment	Upper body segment	Risk assessment of musculoskeletal disorders in real-time with two feedback	A real-time feedback significantly decreased the outcome of both globally as well as locally hazardous RULA values associated with increased risk for musculoskeletal disorders	Joint angle
IMUs	Ranavolo et al. (2017) [174]	20 M	Lifting task	All body	Biomechanical risk assessment using kinematic parameters	Kinematic indices (Lifting Anergy Consumption) were proved to be significantly growing with the LI, discriminating all the risk condition pairs and well correlating with forces that determine injuries at the L5-S1 joint	Joint angles, Center of Mass, Mechanical Energy
IMUs and sEMG	Peppoloni et al. (2016) [3]	8 M, 3 F	Real-life operations of super-market cashiers	Upper limbs	The system exploits IMU to reconstruct the upper limb posture, modeled as a 7- degrees of freedom kinematic chain. sEMG sensors are used to assess forearm flexor muscles strain	The system was capable of autonomously segmenting the cycles and giving a score for each cycle	Joint angles, sEMG
IMUs and sEMG	Brandt et al. (2018) [173]	26 M	Lifting task	Trunk	To classify lifting activities into low and high risk categories based on sEMG and trunk inclination measurements	Lifting tasks were characterized by a feature vector composed of either the 90th, 95th or 99th percentile of sEMG activity level and trunk inclinations during the task applying a linear discriminant analysis and a threshold scheme to classify the lifting tasks with an accuracy of 65.1–65.5%	Trunk inclination, sEMG
Dynamometer and sEMG	Ranavolo et al. (2017) [58]	7 M, 8 F	Manual handling of low loads at high frequency	Upper limbs	To assess the muscle fatigue of the flexor muscles before and after four levels of simulated manual handling of low loads at high frequency; to analyze the calculated fatigue indices to understand whether they correctly classify the risk; to correlate calculated fatigue indices to the muscle behaviors during the execution of the dynamic work task	Fatigue index calculated from the brachioradialis was sensitive to the interaction among risk classes, session and gender	Myoelectric manifestation of muscle fatigue
Hand-held dynamometer	Cadogan et al. (2011) [194]	23 M, 17 F	Active and passive shoulder motion	Shoulder	To establish the reliability of measures of shoulder range of motion (ROM) and muscle force	Active ROM (flexion) demonstrated high levels of both intra- and interexaminer reliability. Passive ROMs and isometric force peaks shown acceptable levels of intraexaminer reliability	Range of motion and muscle force
Grip dynamometer and sEMG	Blackwell et al. (1999) [205]	18 M	Isometric and submaximal gripping contractions	Flexor digitorum superficialis muscle	To investigate the effect of grip span on isometric grip force and fatigue of the flexor digitorum superficialis muscle during sustained voluntary contractions	Fatigue of flexor digitorum superficialis did not change as a function of grip size. Middle grip sizes allowed for greater absolute forces than the small or large size. When contractions are at 60–65% MVC and the muscle is allowed to fatigue, grip size may be less infuential than when maximal absolute force is required	Myoelectric manifestation of muscle fatigue
Dynamometer and sEMG	Roman-Liu et al. (2004) [182]	10 M	Exerting maximal force and tests under specific load conditions	Arm and hand muscles	To discriminate fatigue of upper limb muscles depending on the external load, through the development and analysis of a muscle fatigue index	External loads induced modifications in the fatigue of the biceps brachii caput breve, extensor carpi radialis brevis, and flexor carpi ulnaris muscles	Myoelectric manifestation of muscle fatigue
sEMG and electrogoniometers	Granata and Marras (2000) [57]	10 M	Lifting task	Trunk extensors and flexors muscles	To evaluate whether increased biomechanical stability associated with antagonistic co-contraction was capable of stabilizing the related increase in spinal load	Coactivation was associated with a 12% to 18% increase in spinal compression and a 34% to 64% increase in stability. Spinal load and stability increased with trunk flexion	Muscle coactivation, spinal load and stability
sEMG	Sundelin (1993) [166]	12 F	Repetitive arm work continuously without pauses and with pauses	Trapezius and infraspinatus muscles	Fatigue assessment in shoulder and neck muscles during continuous work and during work with organized pause activities	Muscle fatigue with a decrease in the mean power frequency and an increase in root mean square amplitudes was found both during continuous work and work with pause activities. The muscle fatigue was less pronounced when pause activities were introduced into the work. Fatigue patterns were lower during the second hour, indicating adaptation to the work task and work pace. The ratings of perceived exertion and discomfort were similar during work with and without pauses and were higher during the second hour of work	Myoelectric manifestation of muscle fatigue
sEMG	Sundelin and Hagberg (1992) [165]	6 F	Repetitive arm work for 1 h	Trapezius and infraspinatus muscles	Fatigue assessment in shoulder and neck muscles during work paced by the methods–time measurement system	Muscle fatigue with a decrease in the mean power frequency and an increase in root mean square amplitudes was found	Myoelectric manifestation of muscle fatigue
sEMG	Hansson et al. (1992) [167]	33 F	Static endurance test	Trapezius and deltoid muscles	Muscular fatigue assessment during a standardized isometric endurance test in women with a static workload, with and without neck/shoulder disorders	The endurance time for a group of women in industrial work with repetitive short cycled work tasks who were diagnosed with neck/shoulder disorders was significantly shorter than for a group with the same work, but without neck/shoulder disorders and shorter than for a control group. There were no significant differences in muscle fatigue between the three groups considered	Myoelectric manifestation of muscle fatigue
sEMG	Mathiassen and Winkel (1996) [164]	8 F	Assembly task with different combinations of work pace (120 or 100 according to the methods-time measurement system, MTM), break allowance and duration of the working day	Trapezius muscle	Fatigue assessment in shoulder and neck muscles in different industrial assembly task	During 6 h of work at 120 MTM the EMG amplitude from the upper trapezius muscle increased by about 11%, the EMG zero crossing rate decreased by about 2.5%, and perceived fatigue increased. When work pace was reduced to 100 MTM, the upper trapezius EMG amplitude decreased by 20% and became less variable, perceived fatigue decreased and shoulder tenderness was reduced by about 5%. Added breaks, whether active or passive, had no apparent effects on upper trapezius load during work or on physiological responses	Myoelectric manifestation of muscle fatigue
sEMG	Cabeças (2007) [204]	1 M, 19 F	Cleaning activities	Wrist flexor and extensor muscles	A modified application of the Strain Index method, in evaluation of effort-related variables in cleaning activities	EMG data were found to be a useful alternative to observational methods. The most critical cleaning activities and that with comparatively lower risk to distal upper extremity disorders were individuated	sEMG data (time, intensity, frequency of efforts)
sEMG	Bosch et al. (2009) [183]	5 M, 5 M	Assembly task (construction and break down a tower)	Trapezius muscle	To determine whether muscle fatigue develop in the upper trapezius muscle in two assembly tasks involving contractions of different low-intensity levels and whether these indications of fatigue are homogeneously distributed across different muscle parts	Recordings during task and test showed a significant decrease in the mean power frequency, at both intensity levels while the amplitude remained constant. Significantly different temporal patterns were found for the mean power frequency decrease. No differences in manifestations of muscle fatigue development were found between different parts of the muscle	Myoelectric manifestation of muscle fatigue
sEMG	de Looze et al. (2009) [162]	-	Repetitive low-level force activities	Shoulder muscles	Review of studies on objectively measurable fatigue related changes in time in low-level force activities	Electromyographic manifestations of fatigue in the trapezius muscle appear in low-force activities like light manual work and assembly when the intensity level is about 15–20% MVC. The amplitude increases ranged from 3% to 27%, while the mean power frequency decreases range from 0.9% to 11%. Furthermore, local muscle fatigue seems to occur in some light manual activities and could be considered a risk indicator	Myoelectric manifestation of muscle fatigue
sEMG	Jones and Kumar (2010) [87]	89	Sawmill work (Board-edger operator; Lumber grader; Saw filer; Trim-saw operator)	Wrist flexor and extensor muscles	To examine the agreement between 5 ergonomic risk assessment methods calculated on the basis of quantitative exposure measures and to examine the ability of the methods to correctly classify risk job	RULA and SI were best (correct classification rates of 99 and 97% respectively). The quantitative ACGIH-TLV for monotask hand work and Borg scale were worst (misclassification rates of 86 and 28% respectively)	sEMG and electrogoniometric data
sEMG	Nimbarte et al. (2010) [201]	10 M, 5 F	Lifting tasks carried out at shoulder height in extended, neutral, and flexed neck postures	Neck muscle (sternocleidomastoid and the upper trapezius)	To evaluate physical risk factors (force and posture) associated with neck disorders among construction workers	Increase in the weight lifted increased the activation of the neck muscles. The sternocleidomastoid muscle was most active at the extended neck posture, while the upper trapezius muscle was most active at the flexed neck posture	sEMG data
sEMG	Jia et al. (2011) [202]	19 M, 5 F	Carrying, erecting, lifting and moving tasks	Trunk muscles	Using a model, to predict trunk muscle forces and low back loads during a wide range of panel erection tasks	Reasonable levels of correspondence were found between measured and predicted lumbosacral moments, though predictive ability varied between tasks and rotation planes	Trunk muscle forces and low back loads
sEMG	Draicchio et al. (2012) [188]	10 F	Work activities of supermarket cashiers	Shoulder and trunk muscles	To provide a biomechanical evaluation of cashiers working at a checkout counter before and after a redesign, on the basis of changes induced in time, kinematic and electromyographic variables	The ergonomics intervention (disk wheel) represented a valid aid for reducing biomechanical overload in cashiers and the standing position resulted biomechanically more advantageous. The range of motion values of upper limb and trunk were lowest after the intervention and in the standing position	Range of motion and sEMG data
sEMG	Perez-Duarte et al. (2014) [203]	10 M, 4 F	Conventional laparoscopic and laparoendoscopic single-site surgery	Upper body	To determine inherent risk levels for wrist disorders assessing the degree of arm and back muscle activity as well as spatial configuration of hand and wrist	Muscular activity for trapezius and forearm extensor muscles was significantly lower in conventional laparoscopy compared with single-site approach. A better wrist position was found during laparoendoscopic single-site surgery compared with traditional laparoscopy	sEMG data
sEMG	Ranavolo et al. (2015) [156]	10 M	Lifting task	Trunk extensors and flexors muscles	A method developing for the monitoring of the co-activation of more than two muscles during lifting task	Heavier lifting conditions resulted in higher co-activation values	Muscle coactivation
sEMG	Silvetti et al. (2015) [186]	5 M	Manual handling on a supermarket greengrocery Shelf	Sholuder and trunk muscles	To investigate the effect of different shelf levels and load weights on the workers’ biomechanical load	Shelf level had a significant effect on most of the parameters examined. Weight did not affect the biomechanical load	Ankle joint range of motion and sEMG data
sEMG	Shair et al. (2017) [179]	-	Manual lifting	Arm and trunk muscles	To review the impact of EMG processing in fatigue assessment during manual lifting and to determine the best possible techniques for lifting applications	Bilinear Time-Frequency Distribution (TFD) could perform better than the linear TFD such as Short-Time Fourier Transform (STFT), spectrogram, and Wavelet Transform (WT). Bilinear TFD suffered the cross term effects, that could be removed	sEMG data
sEMG	Le et al. (2017) [65]	-	Isometric loading, Lifting, Isometric quasi-static exertions, motions at different speeds	All body	Understanding of the factors that may influence coactivation and define the necessary variables for a coactivation index that can be used for a variety of tasks	The index appeared to be sensitive to conditions where higher coactivation would be expected. These conditions of higher coactivation included tasks involving higher degrees of control. Precision placement tasks required about 20% more coactivation than tasks not requiring precision, lifting at chest height required approximately twice the coactivation as mid-thigh height, and pushing fast speeds with turning also required at least twice the level of coactivity as slow or preferred speeds	Muscle coactivation
sEMG	Le et al. (2017) [66]	7 M, 10 F	Lifting/lowerin, pushing, and Valsalva maneuvers	Trunk extensors and flexors muscles	To describe the development of an index to assess coactivity for the lumbar spine and test its ability to differentiate between various complex dynamic tasks	Coactivity for dynamic tasks necessitates the understanding of local maxima and minima, different phases of loading, its effect on peak spinal loads, and cumulative responses. It was postulated that a mind–body interaction exists which warrants a continuously defined agonist/antagonist coactivation index sensitive enough to detect those differences	Muscle coactivation
sEMG	Le et al. (2018) [175]	5 M, 7 F	Different combinations of head posture and speed of movement	Neck muscles	Develop a coactivation index for the neck and test its effectiveness with complex dynamic head motions	Complex motions involving twisting and higher speed had higher magnitudes of coactivation than uniplanar motions in the sagittal or lateral plane, which was expected. The coupled motion of flexion and twisting showed four to five times higher coactivation than uniplanar movements	Muscle coactivation
sEMG	Ranavolo et al. (2018) [177]	16 M	Lifting task	Trunk extensors and flexors muscles	sEMG activities of the trunk muscles and forces at the L5-S1 joint to identify sEMG-based indices related to the risk level and to the forces at the L5-S1 joint	sEMG indices were proved to be significantly growing with the LI, discriminating all the risk condition pairs and well correlating with compression and shear forces that determine injuries at the L5-S1 joint	sEMG data, muscle coactivation and forces at the L5-S1 joint
sEMG	Le et al. (2018) [176]	7 M, 10 F	Lifting/lowering, pushing and Valsalva manoeuvres	Trunk muscles	To provide an approach to assess multi-muscle coactivation comparing this index to a coactivation index defined by a biologically assisted lumbar spine model to differentiate between tasks	The EMG-based index was comparable to the index defined by a biologically assisted model. The EMG-based index provided a universal, usable method to assess the neuromuscular effort associated with coactivation for complex dynamic tasks	Muscle coactivation
sEMG	Varrecchia et al. (2018) [178]	10 M	Lifting task	Trunk extensors and flexors muscles	Biomechanical risk assessment using sEMG and neural network	Approaches based on machine-learning techniques and sEMG feature led to an improved biomechanical risk classification	sEMG data in time and frequency domain

## Abbreviations

WMSDs	work-related musculoskeletal disorders
HRC	human-robot collaboration
WLBDs	work-related low-back disorders
UL-WMSDs	upper limb work-related musculoskeletal disorders
RNLE	revised NIOSH lifting equation
KIM-MHO	key indicator method
BIPP	back injury prevention project
PTAI	patient transfer assessing instrument
RAPP	risk assessment of pushing and pulling tool
HAL	hand activity level
SI	strain index
OCRA	occupational repetitive actions
QEC	quick exposure check
OREGE	outil de repérage et d’evaluation des gestes
PATH	posture, activity, tools and handling
RULA	rapid upper limb assessment method
REBA	rapid entire body assessment method
ACGIH TLV	American conference of governmental industrial hygienist’s threshold limit value for mono-task hand work
ART	assessment of repetitive task
ULRA	upper limb risk assessment
MAC	manual handling assessment chart
ALLA	agricultural lower limb assessment
IMUs	inertial measurement units
sEMG	surface electromyography
ARVs	average rectified values
RMS	root mean square
LI	lifting index
LEC	lifting energy consumption
TMCi	time-varying multi-muscle co-activation index
ROM	range of motion
MVC	maximal voluntary contraction
