# Erratum: Alberto, R. et al., Wearable Monitoring Devices for Biomechanical Risk Assessment at Work: Current Status and Future Challenges—A Systematic Review. *Int. J. Environ. Res. Public Health* 2018, 15, 2001

**DOI:** 10.3390/ijerph15112569

**Published:** 2018-11-16

**Authors:** Alberto Ranavolo, Francesco Draicchio, Tiwana Varrecchia, Alessio Silvetti, Sergio Iavicoli

**Affiliations:** 1 Department of Occupational and Environmental Medicine, Epidemiology and Hygiene, INAIL, Via Fontana Candida 1, Monte Porzio Catone, 00078 Rome, Italy; f.draicchio@inail.it (F.D.); al.silvetti@inail.it (A.S.); s.iavicoli@inail.it (S.I.); 2 Department of Engineering, Roma TRE University, Via Vito Volterra 62, 00146 Rome, Italy; tiwana.varrecchia@uniroma3.it

Due to an error during production, the first author’s name of the published paper [1] was incorrect. The corrected name should be Alberto Ranavolo.

The authors would like to apologize for any inconvenience to the readers caused by this error. The article will be updated and the original will remain on the webpage.

## References

[1] Alberto R., Draicchio F., Varrecchia T., Silvetti A., Iavicoli S. (2018). Wearable Monitoring Devices for Biomechanical Risk Assessment at Work: Current Status and Future Challenges—A Systematic Review. Int. J. Environ. Res. Public Health.

